# Pharmacological Inhibition of WEE1 Potentiates the Antitumoral Effect of the *dl922-947* Oncolytic Virus in Malignant Mesothelioma Cell Lines

**DOI:** 10.3390/ijms21197333

**Published:** 2020-10-04

**Authors:** Carmelina Antonella Iannuzzi, Paola Indovina, Iris Maria Forte, Sarah Di Somma, Anna Maria Malfitano, Martina Bruno, Giuseppe Portella, Francesca Pentimalli, Antonio Giordano

**Affiliations:** 1 Cell Biology and Biotherapy Unit, Istituto Nazionale Tumori, IRCCS, Fondazione G. Pascale, I-80131 Naples, Italy; c.iannuzzi@istitutotumori.na.it (C.A.I.); m.forte@istitutotumori.na.it (I.M.F.); 2 Sbarro Institute for Cancer Research and Molecular Medicine, Center for Biotechnology, College of Science and Technology, Temple University, Philadelphia, PA 19122, USA; paola.indovina@icar.cnr.it (P.I.); giordano@temple.edu (A.G.); 3 Institute for High Performance Computing and Networking, National Research Council of Italy (ICAR-CNR), I-80131 Naples, Italy; 4 Dipartimento Scienze Mediche Traslazionali, Università di Napoli “Federico II”, I-80131 Naples, Italy; sarah_ds@hotmail.it (S.D.S.); annamaria.malfitano@unina.it (A.M.M.); 5 Department of Medical Biotechnologies, University of Siena, I-53100 Siena, Italy; st.brunomartina@gmail.com

**Keywords:** malignant mesothelioma, oncolytic adenovirus, *dl922-947*, WEE1, AZD1775, MK-1775, adavosertib, DNA damage response, G2/M checkpoint, apoptosis

## Abstract

Malignant mesothelioma (MM) is a very aggressive asbestos-related cancer, for which no therapy proves to be effective. We have recently shown that the oncolytic adenovirus *dl922-947* had antitumor effects in MM cell lines and murine xenografts. Previous studies demonstrated that *dl922-947*-induced host cell cycle checkpoint deregulation and consequent DNA lesions associated with the virus efficacy. However, the cellular DNA damage response (DDR) can counteract this virus action. Therefore, we assessed whether AZD1775, an inhibitor of the G2/M DNA damage checkpoint kinase WEE1, could enhance MM cell sensitivity to *dl922-947*. Through cell viability assays, we found that AZD1775 synergized with *dl922-947* selectively in MM cell lines and increased *dl922-947*-induced cell death, which showed hallmarks of apoptosis (annexinV-positivity, caspase-dependency, BCL-XL decrease, chromatin condensation). Predictably, *dl922-947* and/or AZD1775 activated the DDR, as indicated by increased levels of three main DDR players: phosphorylated histone H2AX (γ-H2AX), phospho-replication protein A (RPA)32, phospho-checkpoint kinase 1 (CHK1). *Dl922-947* also increased inactive Tyr-15-phosphorylated cyclin-dependent kinase 1 (CDK1), a key WEE1 substrate, which is indicative of G2/M checkpoint activation. This increase in phospho-CDK1 was effectively suppressed by AZD1775, thus suggesting that this compound could, indeed, abrogate the *dl922-947*-induced DNA damage checkpoint in MM cells. Overall, our data suggest that the *dl922-947*-AZD1775 combination could be a feasible strategy against MM.

## 1. Introduction

Malignant mesothelioma (MM) is a very aggressive asbestos-related cancer that arises from the mesothelium lining the body cavities. The most common MM type develops in the pleura, the serous membrane covering the lungs and the chest cavity. Despite the ban on asbestos use in many countries, MM burden is still substantial (over 30,000 MM cases and over 25,000 deaths worldwide in 2018) [1] and is predicted to further rise owing to the long-latency time between exposure and diagnosis [2]. Moreover, asbestos is still used in the developing world and the employment of other asbestos-like fibers that are known to cause MM, such as erionite, is not strictly regulated [3]. The prognosis for patients with MM of the pleura is very poor, with a median survival of approximately 1 year from diagnosis [4]. No current therapeutic strategy is curative: surgery is often challenging and associated with morbidity and mortality [4,5,6] and the only approved first-line chemotherapeutic treatment, consisting of a combination of cisplatin and pemetrexed, has shown limited effects [4,7]. Therefore, there is a great need to identify new targets for the development of effective therapeutic strategies.

Considering that virotherapy-based approaches have recently found a successful application in the clinical setting for different cancer types [8,9,10] and given that MM is a good candidate for this strategy because the pleural location provides direct access for the intra-tumoral injection of the virus [11], we have recently assessed the effects of a selectively replicating oncolytic virus (OV) in MM cell lines and murine xenografts [12]. We used, in particular, the adenovirus *dl922-947*, the efficacy of which has previously been shown by our group and others in cells from different tumors [13,14,15,16,17]. This OV carries a 24-bp deletion in the E1A-Conserved Region 2, which renders viral replication dependent on the inactivation of the retinoblastoma (RB1) pathway [18]. Since disruption of the oncosuppressive RB1 pathway is an almost universal hallmark of human cancers, including MM [19,20], *dl922-947* can kill tumor cells, while sparing normal cells in which the RB1 pathway is functional. We showed that *dl922-947* had antitumor effects in both MM cell lines and xenografts [12]. In particular, *dl922-947* affected cell cycle progression, triggered immunogenic cell death, and reduced the production of pro-angiogenic factors, consistent with the ability of OVs to induce an antitumor immune response [21,22] and a re-shaping of the tumor microenvironment [23,24].

Beyond the above-mentioned mechanisms of *dl922-947* action, the deregulation of multiple cell cycle checkpoints, which accelerates the host cell progression through the cycle, plays an important role for the activity of this OV [25]. Abrogation of these checkpoints results in genomic DNA over-replication and, consequently, in the accumulation of DNA lesions [26,27], which have been found to associate with higher sensitivity to *dl922-947* [27]. However, the virus-induced DNA damage activates the host cell DNA damage response (DDR) signaling, which can counteract the virus action [27,28]. Consistently, we and others showed that inhibitors of crucial factors of the DNA damage signaling and repair, such as ataxia telangiectasia mutated (ATM), checkpoint kinase 1 (CHK1), and poly(ADP-ribose) polymerase (PARP), enhanced the effects of *dl922-947* [26,27,28].

Among the drugs targeting the DDR pathway, AZD1775 (MK-1775, adavosertib), an inhibitor of the tyrosine kinase WEE1, has shown efficacy in sensitizing many cancer types to DNA damaging agents in both preclinical studies and phase I/II clinical trials [29,30,31,32,33,34]. WEE1 is a crucial activator of the G2/M checkpoint, which stalls the cell cycle in response to DNA damage, by phosphorylating and inhibiting cyclin-dependent kinase 1/2 (CDK1/CDK2). WEE1 inhibition leads to G2/M checkpoint override, unscheduled mitotic entry, increased replication stress, subsequent nucleotide starvation, and loss of genomic integrity [30]. G2/M checkpoint abrogation through WEE1 inhibition was originally conceived as a strategy to selectively sensitize cancer cells to DNA damaging agents, given that most human cancers rely on the G2/M checkpoint to detect and repair damaged DNA [35]. Indeed, the G1/S checkpoint is defective in almost all cancers because of the loss of the p53 tumor suppressor. Therefore, tumor cells treated with a WEE1 inhibitor are forced to enter aberrant and lethal mitosis in the presence of DNA damage; conversely, non-neoplastic cells, which retain G1/S checkpoint activity, are unaffected by this treatment. Based on this rationale, many studies focused on the effects of WEE1 inhibition in combination with DNA damaging agents in tumors bearing *TP53* mutations. However, other mechanisms, such as DDR aberrations, nucleotide starvation, replicative stress, and, as more recently found, loss of the *ATRX* chromatin remodeler gene [36] and low phosphatase and tensin homolog (PTEN) expression [37], contribute to sensitize cancer cells to WEE1 inhibition, which, thus, proved monotherapy activity even in *TP53*-wild-type cancer cells [29,30,38]. Moreover, WEE1 inhibition showed efficacy also in combination with inhibitors of other DDR factors, such as PARP [39,40,41,42], CHK1 [29,43,44,45,46,47], and ataxia telangiectasia and Rad3 related (ATR) kinase [48,49,50], and also when combined with different anticancer targeted agents [29,51,52,53,54,55,56,57,58,59,60,61] and immunotherapeutic approaches [29,62,63,64].

We have previously demonstrated that WEE1 inhibition sensitizes MM cells to the DNA-damaging agent cisplatin by forcing them to enter mitosis despite damaged DNA [65], as further confirmed also by others in a more recent study [66]. We have also previously observed that *dl922-947* induces DNA over-replication in MM cells [12], which could be indicative of possible DNA damage generation. In the present study, we found that *dl922-947* induces, indeed, a DDR in MM cells and that WEE1 inhibition through AZD1775 synergizes with *dl922-947* by abrogating the DNA damage checkpoint and increasing cell death. Thus, our data suggest that the combination of these agents could be a feasible strategy against MM.

## 2. Results

### 2.1. AZD1775 Synergizes with dl922-947 in MM Cell Lines

**Figure 1:**
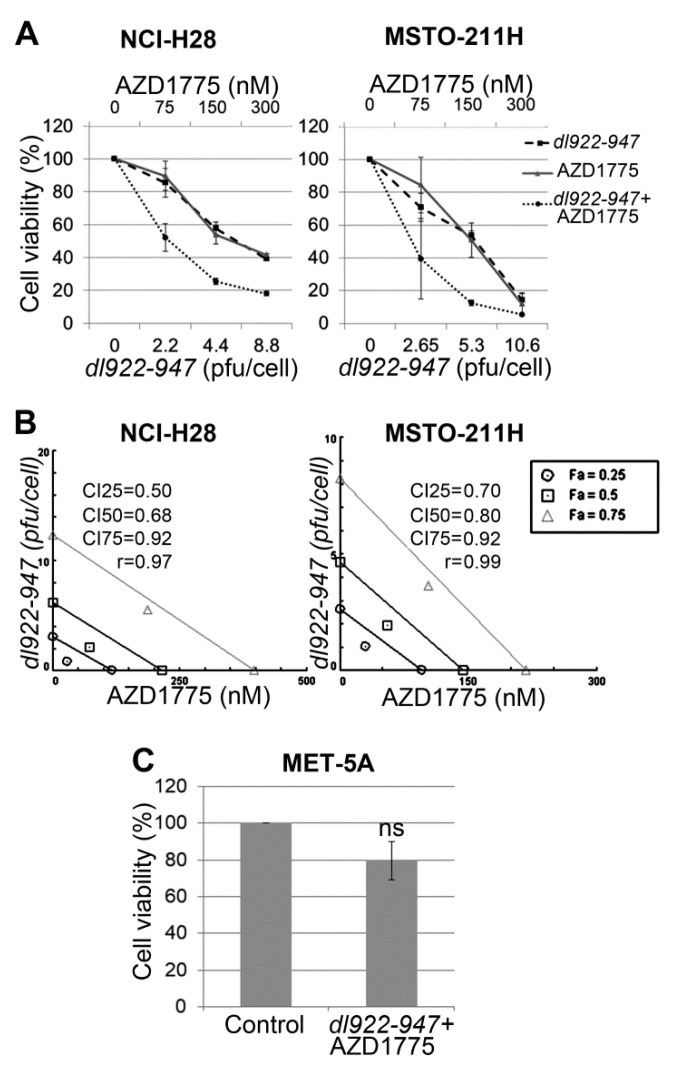
Synergistic effect of *dl922-947*-AZD1775 combination on malignant mesothelioma (MM) cell lines. (**A**) Dose–response curves for *dl922-947* alone, AZD1775 alone, and *dl922-947*-AZD1775 combination in NCI-H28 and MSTO-211H cell lines 5 days after treatment. Results represent the means with standard deviation of 2 independent experiments, each conducted in triplicate, and are expressed as percentages of cell viability calculated with respect to control cells treated with DMSO alone. (**B**) Isobologram analysis to evaluate synergism between *dl922-947* and AZD1775. Isobolograms are derived from the mean values of the dose–response experiments reported in **(A)**, through the CompuSyn software 1.0 (ComboSyn, Inc., Paramus, NJ, USA), at effect levels (Fa, fraction affected) of 25, 50, and 75%. Data points on the line indicate additivity; points below the line indicate synergy; points above the line indicate antagonism. The combination indexes (CIs) at 25, 50, and 75% of cell killing (CI25, CI50, CI75, respectively) and r values are also reported. Combination index (CI) values < 1 indicate synergism. (**C**) Histogram representing MET-5A cell viability analyzed 5 days after *dl922-947*-AZD1775 co-treatment. Cell viability was calculated as a percentage with respect to control cells treated with DMSO alone. Results represent the means ± standard deviation of 3 experiments, each conducted in triplicate. The absorbance values of treated and control cells were subjected to paired Student’s t-test and showed no significant difference (ns, not significant).

To evaluate whether WEE1 inhibition by AZD1775 enhances *dl922-947* efficacy in MM cells, we challenged NCI-H28 and MSTO-211H cell lines for 5 days with the two agents, both alone and in combination at different concentrations in a constant ratio. In particular, the agents were added in 2-fold serial dilutions above and below their 5-day half maximal inhibitory concentration (IC50) values, which were 4.4 and 5.3 pfu/cell of *dl922-947* in NCI-H28 and MSTO-211H, respectively (as we previously reported [12]), and 150 nM of AZD1775 in both cell lines. Cell viability data were obtained through sulforhodamine B (SRB) assay (Figure 1A) and evaluated by isobologram analysis, which showed synergism between AZD1775 and *dl922-947* in both cell lines (Figure 1B).

To rule out possible cytotoxic effects of the *dl922-947*-AZD1775 combination on non-neoplastic cells, we treated MET-5A cell line, derived from normal mesothelium, with this drug combination (at approximately the IC50 value identified in tumor cells). We observed only a slight, not significant toxicity after 5 days of treatment (Figure 1C).

### 2.2. AZD1775 Increases the dl922-947-Induced Cell Death in MM Cells

**Figure 2:**
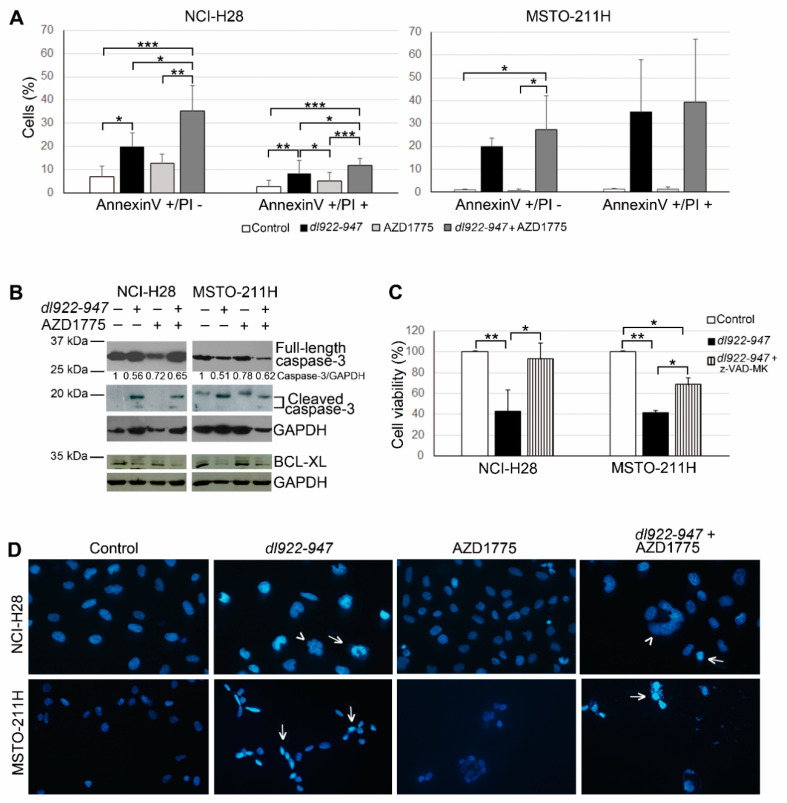
Cell death induction in malignant mesothelioma (MM) cells treated with *dl922-947* and/or AZD1775. (**A**) Histograms report the means with standard deviations of at least three independent experiments representing the percentage of positive cells stained with annexinV–FITC and propidium iodide (PI) 96 h after treatment with *dl922-947* and/or AZD1775 or DMSO, as a control. Statistically significant differences were evaluated by one-way repeated measures ANOVA with Tukey post-test and indicated as follows: * *p* < 0.05, significant; ** *p* < 0.01, very significant; and *** *p* < 0.001, extremely significant. (**B**) Caspase-3 and BCL-XL protein levels were analyzed by Western blotting in NCI-H28 and MSTO-211H cells treated as reported above. The antibody against caspase-3 detects both the full-length protein and the active cleaved form, which are shown separately at different exposure times (the full-length protein bands are shown at a shorter exposure time because they become overexposed at the time necessary for cleaved caspase-3 bands to appear). GAPDH was used as a loading control. Full-length caspase-3 band densities were quantified by densitometric analysis and normalized with the GAPDH band densities. Data are presented as relative values with respect to control values, set at 1. (**C**) Histograms reporting NCI-H28 and MSTO-211H cell viability 96 h after treatment with *dl922-947* alone and in combination with Z-VAD-FMK. Results represent the means with standard deviations of at least 2 independent experiments, each conducted in triplicate, and are expressed as percentages of cell viability calculated with respect to untreated control cells. Statistically significant differences were evaluated by one-way repeated measures ANOVA with Tukey post-test and indicated as follows: * *p* < 0.05, significant; ** *p* < 0.01, very significant. (**D**) Representative fluorescence micrographs of DAPI-stained NCI-H28 and MSTO-211H cells treated with *dl922-947* and/or AZD1775 as reported above. Arrows indicate some of the nuclei with clumped or condensed chromatin; arrowheads indicate some multilobed nuclei or multinucleation.

To assess cell death induction by *dl922-947* and/or AZD1775 in NCI-H28 and MSTO-211H cell lines, we analyzed, through FACS, double staining with annexinV–FITC, which detects an early apoptosis marker, and propidium iodide (PI), which indicates membrane permeabilization in necrotic/late apoptotic cells. Ninety-six hours after treatment with the two agents at their IC50 values, we observed an increase in the percentage of both annexinV-positive–PI-negative cells (indicative of early apoptosis) and annexinV-positive–PI-positive cells (indicative of late apoptosis/necrosis), which was higher after *dl922-947* and AZD1775 co-treatment than after *dl922-947* infection alone (Figure 2A). Conversely, treatment with AZD1775 alone did not induce a significant increase in annexinV positivity.

We also evaluated the activation of the apoptosis marker caspase-3 in NCI-H28 and MSTO-211H cells treated as above, by Western blotting analysis; we observed that *dl922-947*, both alone and in combination with AZD1775, induced an increase in the active cleaved caspase-3 levels and a concurrent slight decrease in the full-length protein (Figure 2B). To confirm the role of caspase activation in *dl922-947*-induced cell death, we co-treated NCI-H28 and MSTO-211H cells with *dl922-947* and the pan-caspase inhibitor Z-VAD-FMK. We found that these cells had a significantly higher cell viability than cells treated with *dl922-947* alone (Figure 2C).

Considering that, among BCL-2 family members, the anti-apoptotic protein BCL-XL was previously found to be particularly important for survival of MM cells, including NCI-H28 and MSTO-211H [67], we analyzed by Western blotting its expression in these cell lines treated with *dl922-947* and/or AZD1775, as described above. We observed decreased BCL-XL levels in both cell lines upon treatment with *dl922-947* and *dl922-947-*AZD1775 combination (Figure 2B).

Moreover, DAPI staining showed that NCI-H28 and MSTO-211H cells, treated with *dl922-947* and its combination with AZD1775, had clumped or condensed chromatin, which is compatible with the apoptotic cell death. These cells also had other alterations in nuclear morphology (multilobed nuclei, multinucleation), which could be in line with aberrant mitoses (Figure 2D). Conversely, cells treated with AZD1775 alone had uncondensed and homogeneously distributed chromatin, similar to untreated control cells. This is consistent with the lack of a significant increase in apoptotic markers that we observed in AZD1775-treated cells.

### 2.3. AZD1775 Inactivates the DNA Damage Checkpoint Induced by dl922-947 in MM Cells

To study the molecular mechanism whereby WEE1 inhibition by AZD1775 sensitizes MM cells to *dl922-947*, we analyzed the effects of these agents on the WEE1 direct substrate, CDK1, and also on crucial factors of the DDR signaling. In particular, we analyzed phosphorylated histone H2AX (γ-H2AX), which is a well-known marker of double-strand breaks, and replication protein A (RPA) and CHK1, which have previously been implicated in the DDR pathway induced by *dl922-947* [27,28] or AZD1775 [44,68,69,70,71,72,73] in other cancer cell types.

RPA is a heterotrimeric protein complex, consisting of RPA70, RPA32, and RPA14 subunits, which acts as a sensor of DNA damage and replication stress, by associating with single-stranded DNA [74]. DNA damaging agents induce RPA32 N-terminus phosphorylation, which is involved in the checkpoint response mediated by the ATR kinase, leading to CHK1 phosphorylation and activation [74]. CHK1 transduces the damage signals to a variety of effectors, resulting in cell cycle checkpoint activation, cell cycle arrest, DNA repair, or cell death [75].

**Figure 3:**
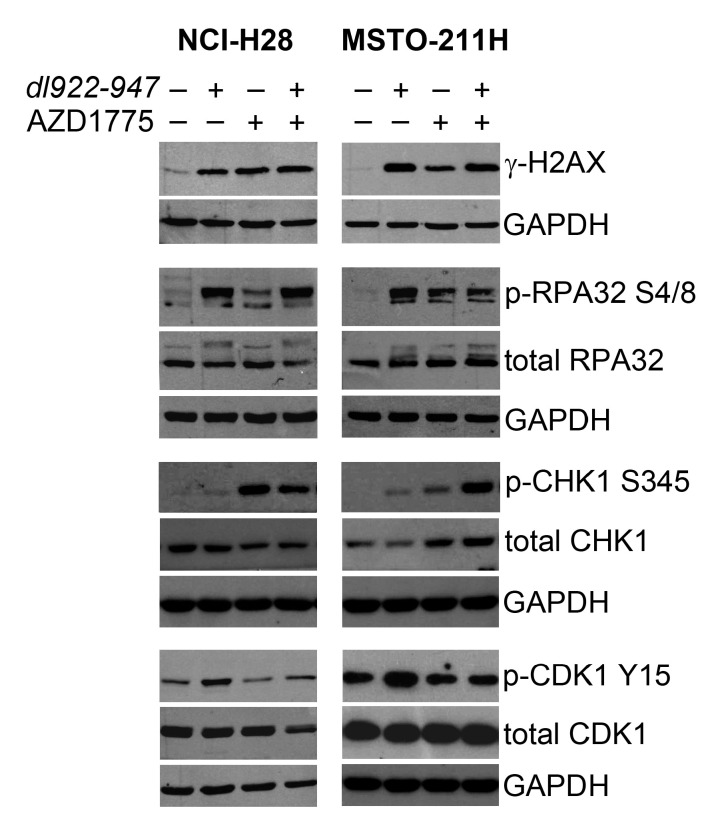
Effect of *dl922-947* and AZD1775 on the activation of cyclin-dependent kinase 1 (CDK1) and DNA damage signaling factors in malignant mesothelioma (MM) cell lines. NCI-H28 and MSTO-211H cell lines were treated with *dl922-947* and AZD1775, both alone and in combination, and analyzed through Western blotting for the following factors: phosphorylated histone H2AX (γ-H2AX); phospho-replication protein A (RPA)32 Ser 4/Ser 8 and total RPA32; phospho-checkpoint kinase 1 (CHK1) Ser 345 and total CHK1; phospho-CDK1 Tyr 15 and total CDK1. DMSO was added to untreated control cells. An anti-GAPDH antibody was used for loading control.

Through Western blotting, we observed that *dl922-947* and AZD1775, both alone and in combination at their respective IC50 values, induced the expression of γ-H2AX, phospho-RPA32 Ser 4/Ser 8, and phospho-CHK1 Ser 345 in both MM cell lines, which is indicative of DDR activation (Figure 3). Consistently, we observed that *dl922-947* increased the levels of the inactive Tyr-15-phosphorylated form of CDK1, denoting G2/M checkpoint activation (Figure 3). As expected, this increase in phospho-CDK1 was suppressed by AZD1775 (Figure 3), showing that this inhibitor can effectively prevent the WEE1-mediated phosphorylation and inactivation of CDK1, which could thereby abrogate the *dl922-947*-induced DNA damage checkpoint in MM cell lines.

## 3. Discussion

MM is a very aggressive asbestos-associated cancer for which at present there is no curative modality. Although significant efforts have been made to reduce occupational exposure to asbestos, MM incidence is expected to rise because of the long-latency time between exposure and diagnosis [2]. Moreover, asbestos and other mineral fibers that are known to cause MM are still employed in some countries [3]. Therefore, there is an urgent need to identify new therapeutic avenues.

Considering that MM is a good candidate for innovative virotherapy-based approaches because the pleural location provides direct access for the intra-tumoral injection of the virus [11], we have recently analyzed the effects of the oncolytic adenovirus *dl922-947* in MM cells. The replication of this OV, bearing a deletion in the RB1 binding site of the E1A region, is dependent on RB1 inactivation [18], which occurs very frequently in MM. Indeed, although RB1 mutations are extremely rare in this cancer type (COSMIC, the Catalogue of Somatic Mutations in Cancer, http://cancer.sanger.ac.uk), the homozygous deletion of the cyclin-dependent kinase inhibitor 2A (*CDKN2A)* locus, which results in RB1 functional inactivation, is one of the most common mutations in MM cells [19,20], including the NCI-H28 and MSTO-211H cell lines under study (COSMIC). We have found that *dl922-947* has antitumor effects in both MM cell lines and xenografts by affecting cell cycle progression, triggering immunogenic cell death, and reducing the production of pro-angiogenic factors [12].

In particular, we have observed that *dl922-947* induces DNA over-replication in MM cells, which can generate DNA damage. Since the ability of *dl922-947* to induce DNA lesion accumulation associates with its efficacy [27] and given that DDR inhibitors can favor this mechanism of action [26,27,28], in the present study, we analyzed whether the abrogation of the G2/M DNA damage checkpoint through the WEE1 inhibitor AZD1775 enhanced MM cell sensitivity to *dl922-947*. Through cell viability assays and isobologram analysis, we found that AZD1775 synergized with *dl922-947* in MM cell lines.

We previously reported that both *dl922-947* [12] and AZD1775 [65] sensitized MM cells to cisplatin, the drug currently used in MM chemotherapy. The comparison of the present data with our previous findings shows that, at the same treatment time and doses, *dl922-947* affects cell viability similarly or even more efficaciously when combined with AZD1775 than when combined with cisplatin in MSTO-211H and NCI-H28 cells, respectively. Therefore, the *dl922-947*-AZD1775 co-treatment seems worthy of further investigations; in particular, its efficacy should be evaluated in animal models, also in comparison with that of other mono- and combination therapies.

We also found that *dl922-947*-AZD1775 combination did not significantly affect the viability of the normal mesothelial cell line MET-5A, thus again encouraging further testing of this strategy.

To analyze cell death induction, we performed the annexinV assay, which detects a well-known apoptosis marker, namely the exposure of phosphatidylserine to the cell surface. We observed an increase in the percentage of annexinV-positive cells, which was higher after the combination treatment with *dl922-947* and AZD1775 than after the treatment with *dl922-947* alone. Moreover, upon treatment with *dl922-947*, both alone and in combination with AZD1775, we also observed the activation of the apoptotic marker caspase-3. We confirmed the caspase-dependency of the *dl922-947*-induced cell death by using the pan-caspase inhibitor Z-VAD-FMK, which significantly increased cell viability. We also observed a decreased expression of the anti-apoptotic protein BCL-XL in NCI-H28 and MSTO-211 cells following treatment with *dl922-947* and *dl922-947*-AZD1775 combination. This is in line with previous data indicating a crucial role of BCL-XL in regulating cell death of these cell lines [67]. Furthermore, we observed a nuclear morphology compatible with the apoptotic cell death upon treatment with *dl922-947* and its combination with AZD1775. Conversely, in previous studies on ovarian cancer cells, classical hallmarks of apoptosis were not detected after treatment with *dl922-947* or other oncolytic adenoviral mutants, which have been suggested to induce a different type of programmed cell death [76,77]. However, it seems plausible that *dl922-947* could trigger different cell death processes in diverse cancer cell types, although further studies are necessary to understand the exact mode of cell death induced by this OV in MM. In particular, the characterization of the whole process requires a more thorough definition of the factors involved and the extension of the analysis to a wider set of MM cell lines, taking into account their possible dependency on different mechanisms to control cell death.

We observed that AZD1775 and *dl922-947*, both alone and in combination, activated the DDR pathway, as indicated by the increase in expression of γ-H2AX, phospho-RPA32 Ser 4/Ser 8, and phospho-CHK1 Ser 345, and in line with what was previously found in other cancer cell types [27,28,44,68,69,70,71,72,73]. Moreover, we observed that *dl922-947* increased the levels of the inactive Tyr-15-phosphorylated form of CDK1, which is a surrogate marker of G2/M checkpoint activation. This increase in phospho-CDK1 was suppressed by AZD1775. Thus, this molecule seems indeed to abrogate the *dl922-947*-induced DNA damage checkpoint in MM cell lines by effectively preventing the WEE1-mediated phosphorylation and inactivation of CDK1.

G2/M checkpoint abrogation through WEE1 inhibition in combination with DNA-damaging agents has initially been suggested to be mainly effective on cancer cells bearing mutations in the key G1/S checkpoint regulator *TP53* since these cells rely on the G2/M checkpoint to detect and repair damaged DNA [35]. However, this approach proved to be successful also in preclinical studies on *TP53* wild-type MM cells most likely because of the occurrence in these cells of G1/S checkpoint inactivation through mechanisms other than direct *TP53* mutation [65,66]. In particular, the homozygous deletion of the *CDKN2A* locus, which, as stated above, is very common in MM cells, results in the functional inactivation of both the key tumor suppressors controlling the G1/S checkpoint, p53 and RB1 [20]. Consistent with these observations, in this study, we found that the *dl922-947*-AZD1775 combination was effective in NCI-H28 and MSTO-211H cell lines, which, while expressing wild-type *TP53* [78], both carry a homozygous deletion of the tumor suppressor locus *CDKN2A* (COSMIC, http://cancer.sanger.ac.uk). Thus, disruption of crucial tumor-suppressive pathways, which largely underlies MM development [7], seems to offer therapeutic opportunities for this cancer. Indeed, two main anticancer strategies can be used to target tumor suppressors: the first aims to exploit their loss, whereas the second aims to reactivate their function [79,80,81]. Accordingly, in our previous preclinical studies on MM, we have shown the potential anticancer efficacy of strategies based on the reactivation of the oncosuppressive functions of p53 [78], RBL2/p130, which is another crucial member of the RB family [82], and p27, which is a cell cycle inhibitor co-regulated with RBL2/p130 [82,83]; conversely, in the present study, we used agents that exploit tumor suppressor inactivation for their selective anticancer action: the oncolytic adenovirus *dl922-947*, which depends on RB1 inactivation for its replication, and AZD1775, which is considered to be mainly effective in cancer cells defective in G1/S checkpoint regulators.

Similar combinations between *dl922-947* and other DDR inhibitors were analyzed in previous studies on different cancer types by both our group and others [26,27,28]. Here, we combined *dl922-947* with AZD1775 based on our previous observation of the efficacy of WEE1 inhibition in sensitizing MM cells to the DNA-damaging agent cisplatin [65]. Moreover, in a recent kinome-wide CRISPR/Cas9 knockout screening, which has identified several kinases whose deficiency improves chemotherapy efficacy in MM, WEE1 knockout has proved to induce the most significant effect [66].

Although different DDR inhibitors have partially overlapping effects, they have also unique and complementary modes of action, which are responsible for their synergy [68,70,72,84,85,86]. Thus, rational combinations of these inhibitors could be useful to overcome or prevent resistance [30]. Recent studies have shown that triplet regimens consisting of chemotherapy plus WEE1 and CHK1 inhibitors could be particularly efficacious [72,87]. Possible triple treatments combining the oncolytic adenovirus *dl922-947* with DDR inhibitors deserve future investigation.

In conclusion, we found that WEE1 inhibition through AZD1775 sensitizes MM cells to *dl922-947* by abrogating the DNA damage checkpoint induced by the virus. Although the *dl922-947*-AZD1775 combination has yet to be assessed in animal models, this selective anticancer strategy, which depends on oncosuppressive pathway disruption, could be an optimal approach against a tumor, such as MM, that mainly develops through the loss of tumor suppressor functions.

## 4. Materials and Methods

### 4.1. Cell Cultures and Adenovirus Preparation

NCI-H28 and MSTO-211H mesothelioma cell lines and MET-5A mesothelial cells were purchased from American Type Culture Collection (ATCC; Manassas, VA USA). NCI-H28 and MSTO-211H cells were grown in RPMI-1640 supplemented with 10% fetal bovine serum (FBS), 1% penicillin-streptomycin, and 1% glutamine. MET-5A cells were grown in Medium 199 with 10% FBS, 0.5% penicillin-streptomycin, 1% glutamine, and 3.3 nM epidermal growth factor, 400 nM hydrocortisone, and 870 nM insulin. All cell culture reagents were obtained from Sigma-Aldrich (St Louis, MO, USA), and cells were maintained in a humidified incubator set at 37 °C and 5% CO_2_. Cells were routinely tested with the PlasmoTest™ Mycoplasma Detection kit (Invivogen, San Diego, CA, USA) for the presence of mycoplasma, which was eradicated with Plasmocin™ Mycoplasma Elimination Reagent (Invivogen), when necessary. *Dl*922-947 was expanded in the human embryonic kidney cell line HEK-293 (ATCC), purified, stored, and quantified in 8.3 × 10^8^ pfu/mL viral stocks, as previously described [12].

### 4.2. Drug Combination, Sulforhodamine B (SRB) Assay, and Synergism Analysis

MM cells were seeded in 96-well plates 24 h before treatment with *dl*922-947 and AZD1775 (MK1775, purchased from Axon Medchem), both alone and in combination at various concentrations in a constant ratio. Five days after treatment, cells were fixed with 50% (*v*/*v*) trichloroacetic acid and stained with 0.4% (*w*/*v*) SRB in 1% (*v*/*v*) acetic acid, following the manufacturer’s instructions. Synergism, additivity, or antagonism were determined through isobologram analysis using the CompuSyn software 1.0 (ComboSyn, Inc., Paramus, NJ, USA). Combination index (CI) values were also calculated by the CompuSyn software, which uses the Chou–Talalay method. CI < 1 indicates synergism, CI = 1 additivity, and CI > 1 antagonism. The r value represents the linear correlation coefficient of the median effect plot, which indicates the conformity of the data to the mass action law.

### 4.3. Apoptosis Analysis

Apoptosis was assessed through flow cytometric analysis (BD FACSCalibur, Becton Dickinson BD Biosciences, Franklin Lakes, NJ, USA) of MM cells treated for 96 h with *dl922-947* and/or AZD1775 at their IC50 values and stained with annexinV–FITC and propidium iodide (AnnexinV-FITC Kit, Biolegend, San Diego, CA, USA) according to the manufacturer’s instructions.

To evaluate whether the *dl922-*947-induced cell death was caspase-dependent, the cells were treated with *dl922-947* alone and in combination with the pan-caspase inhibitor Z-VAD-FMK (R&D Systems, Minneapolis, MN, USA) at the concentration of 100 µM. Ninety-six hours after treatment, cell viability was evaluated by MTS assay (CellTiter 96^®^ AQueous One Solution Cell Proliferation Assay, Promega, Madison, WI, USA), following the manufacturer’s instructions.

### 4.4. DAPI Staining

NCI-H28 and MSTO-211H cells were grown on coverslips and treated with *dl*922-947 and AZD1775, both alone and in combination. Ninety-six hours after treatment, the cells were fixed in 3% paraformaldehyde for 10 min and permeabilized with 0.5% triton-X 100 for 10 min. Samples were then blocked in 1% BSA for 10 min. The coverslips were mounted using the ProLong Gold Antifade Reagent with DAPI (Life Technologies, Carlsbad, CA, USA). Images were obtained using the Nikon Eclipse E600 microscope (Nikon, Minato, Tokyo, Japan).

### 4.5. Western Blotting Analysis

For total protein extraction, cells were lysed on ice for 30 min in a buffer consisting of 50 mM Tris-HCl pH 7.5, 1 mM EDTA pH 8.0, 150 mM NaCl, 1% NP-40, supplemented with protease and phosphatase inhibitor cocktails (Roche, Basilea, Switzerland). The protein samples were resolved by SDS-PAGE and blotted onto nitrocellulose membranes, which were then incubated with antibodies against: ɣ-H2AX (Cat. #05-636) from Merk Millipore, Burlington, MA, USA; phospho-RPA32 Ser 4/Ser 8 (Cat. #A300–245A) and RPA32 (Cat. #A300–244A) from Bethyl Laboratories, Montgomery, TX, USA; phospho-CHK1 Ser 345 (Cat. #2348), CHK1 (Cat. #2360), phospho-CDK1 Tyr15 (Cat. #4539), CDK1 (Cat. #9116S), and caspase-3 (Cat. #9662S) from Cell Signaling Technologies, Danvers, MA, USA; BCL-XS/L (Cat. #sc-1041) and GAPDH (Cat. #sc-25778) from Santa Cruz Biotechnology, Santa Cruz, CA, USA. After incubation with horseradish peroxidase-conjugated secondary antibodies, signals were detected through ECL (Amersham Biosciences, GE Healthcare, Little Chalfont, UK). The chemiluminescent images were analyzed by ImageQuant LAS 500 (GE Healthcare, Little Chalfont, UK).

### 4.6. Statistical Analysis

Results were expressed as means ± standard deviation and derived from at least two independent experiments. Statistical analyses were performed through one-way repeated measures ANOVA with Tukey post-test (to compare multiple matched groups) and through paired Student’s t test (to compare two matched groups) using the GraphPad Software 5.01 (GraphPad Software, San Diego, CA, USA). *p* values < 0.05 were considered as significant.

## Abbreviations

ATM	Ataxia telangiectasia mutated
ATR	Ataxia telangiectasia and Rad3 related
CDK	Cyclin-dependent kinase
CDKN2A	Cyclin-dependent kinase inhibitor 2A
CHK1	Checkpoint kinase 1
CI	Combination index
COSMIC	Catalogue of Somatic Mutations in Cancer
DDR	DNA damage response
Fa	Fraction affected
FBS	Fetal bovine serum
IC50	Half maximal inhibitory concentration
MM	Malignant mesothelioma
OV	Oncolytic virus
PARP	Poly(ADP-ribose) polymerase
PI	Propidium iodide
RB	Retinoblastoma
RPA	Replication protein A
SRB	Sulforhodamine B
γ-H2AX	Phosphorylated histone H2AX
